# Role of tumor mutational burden in patients with urothelial carcinoma treated with immune checkpoint inhibitors: a systematic review and meta-analysis

**DOI:** 10.3389/fimmu.2025.1592761

**Published:** 2025-05-26

**Authors:** Zhe Wang, Danxue Huang, Su Li, Liyuan Ke

**Affiliations:** ^1^ Department of Urology, Cancer Hospital of China Medical University, Liaoning Cancer Hospital and Institute, Shenyang, China; ^2^ Department of Pharmacy, Cancer Hospital of China Medical University, Liaoning Cancer Hospital and Institute, Shenyang, China

**Keywords:** tumor mutation burden, immune checkpoint inhibitor, urothelial carcinoma, overall survival, progression-free survival, objective response rate, meta-analysis

## Abstract

**Background:**

The predictive value of tumor mutation burden (TMB) on the efficacy of immunotherapy has been confirmed in multiple cancer types in previous studies. For urothelial carcinoma (UC) patients treated with immune checkpoint inhibitors (ICIs), whether TMB is a suitable biomarker to predict the benefit of ICIs remains a matter of much debate. We conducted this meta-analysis to evaluate the role of TMB in patients with UC treated with ICIs.

**Methods:**

Two investigators independently searched the literature, screened eligible studies, extracted valid data, and scored quality assessments. Meta-analyses of the effect size hazard ratio (HR) for overall survival (OS) and progression-free survival (PFS), and effect size odds ratio (OR) for objective response rate (ORR) were performed and visualized with forest plots using the STATA14.0 software. The statistical difference in benefit from ICIs for UC patients between the high TMB group and the low TMB group was significant when the *p*-value <0.05. Sensitivity analysis and publication bias further verified the stability and reliability of statistical results.

**Results:**

A total of 2,499 patients from 14 studies were included in this meta-analysis. The results indicated that UC patients with high TMB showed significantly longer OS and PFS than those with low TMB after ICI treatment (OS: HR 0.69, 95% CI 0.62, 0.76, *p* < 0.05; PFS: HR 0.67, 95% CI 0.59, 0.76, *p* < 0.05). The high TMB group exhibited a superior response to ICIs than the low TMB group, with no significant difference (OR 1.64, 95% CI 0.94, 2.86, *p* = 0.08). The results were stable and reliable, with no publication bias.

**Conclusions:**

This meta-analysis demonstrated that UC patients with high TMB exhibited significantly longer survival than those with low TMB after ICI treatment. TMB may be a favorable predictor for UC immunotherapy in future clinical practice.

**Systematic Review Registration:**

https://www.crd.york.ac.uk/prospero/, identifier CRD42025642602.

## 1 Introduction

With nearly 600,000 new cases and more than 200,000 deaths each year, urothelial carcinoma (UC) is a common malignancy worldwide (1). For decades, chemotherapy involving platinum has played a pivotal role in the standardized therapy for advanced UC (2). With the emergence of novel agents, immune checkpoint inhibitors (ICIs) provide more options for patients, such as first-line treatment, second-line treatment with disease progression during or following chemotherapy, and maintenance treatment after first-line platinum-containing chemotherapy (3–5). The phase 3 trial CheckMate-901 demonstrated that patients treated with gemcitabine plus cisplatin with nivolumab obtained more benefits of progression-free survival (PFS) and overall survival (OS) than those treated with cisplatin-based chemotherapy, providing evidence supporting the approval of nivolumab as first-line treatment (3). The efficacy of pembrolizumab in locally advanced UC with disease progression on or after platinum-containing chemotherapy was investigated in the KEYNOTE-045 study (4). The study demonstrated statistically significant improvements in objective response rate (ORR) for patients randomized to pembrolizumab compared to chemotherapy. The survival benefit from the maintenance treatment with avelumab in the JAVELIN trial was proven in patients with locally advanced UC who were in stable condition after first-line chemotherapy (5).

The meta-analysis assessing the efficacy of ICIs as first-line therapy demonstrated that ICIs could be used as a safe and viable first-line treatment option for advanced or metastatic UC who are ineligible for platinum-based chemotherapy (6). A network meta-analysis in advanced or metastatic UC reported that ICI plus chemotherapy as first-line treatment resulted in longer PFS and higher ORR compared with chemotherapy, but ICI alone as first-line treatment did not provide additional benefit (7). Nevertheless, the abridged Cochrane review confirmed that immunotherapy provided a survival benefit compared to chemotherapy as a second-line treatment, but not as a first-line one (8). The updated analyses demonstrated that adjuvant ICIs significantly improved disease-free survival (DFS) and overall survival in patients with high-risk muscle-invasive urothelial carcinoma compared with the placebo/observation group (9). The meta-analysis of 10 randomized controlled trials showed that UC patients treated with atezolizumab as a monotherapy or in combination with chemotherapy had a significantly longer OS compared to those who were treated with a placebo (10). A study of high-risk muscle-invasive urothelial carcinoma (MIUC) compared indirectly the efficacy of pembrolizumab, nivolumab, and atezolizumab as adjuvant treatments, and the results showed that there was no significant difference in DFS among them (11). An observational multicenter study of metastatic UC patients treated with avelumab or pembrolizumab showed no significant differences between the two agents (12).

To aid in the therapeutic judgment of ICIs, more predictive biomarkers have become the focus of exploratory research in tumor genomics. Although programmed cell death ligand 1 (PD-L1) status may be associated with response to ICIs, some PD-L1-negative patients have a response, whereas PD-L1-positive patients do not. Tumor mutational burden (TMB), defined as the quantity of mutations existing in the tumor genome, may be a promising biomarker to predict the efficacy of immunotherapy. Tumor cells with a high mutational burden are likely to produce highly immunogenic neoantigens (13). Tumor-derived mutated proteins are internalized and processed by antigen-presenting cells into antigenic peptides and then presented to CD8+ T cells, which are regulated by cytotoxic T lymphocyte antigen 4 (CTLA-4). CD8+ T cells are able to recognize tumor cells presenting the same antigen and induce apoptosis, which is regulated by programmed cell death protein 1 (PD-1) and PD-L1. ICIs targeting CTLA4, PD-1, and PD-L1 prevent tumors from evading immune surveillance. The hypothesis of high TMB patients expressing better efficacy of ICIs has been investigated across various cohorts (14).

Over the last few years, several studies have demonstrated that higher TMB is associated with improved outcomes in metastatic UC patients treated with ICIs (15, 16). The IMvigor210 study of efficacy and safety analysis of atezolizumab in patients with cisplatin-ineligible locally advanced or metastatic UC conducted exploratory biomarker assessments and showed that mutation load was associated with OS. TMB was binned into quartiles, and patients with the highest mutation load (quartile 4) had significantly longer survival than those in quartiles 1–3 (15). Targeted genomic profiling of 315 cancer-related genes examined in UC patients showed that the median mutation load of responders to atezolizumab was located at a higher level compared with non-responders, and this relationship was independent of other gene expression (16). As a milestone, a TMB level above 10 Mut/Mb was set as the standard for histology-agnostic approval of pembrolizumab in all solid tumors based on data from the Keynote-158 trial (17).

To date, there has been no definitive conclusion indicating the role of TMB in patients with UC treated with ICIs. We have contributed to this first meta-analysis to evaluate the prognostic significance of TMB in UC patients treated with ICIs.

## 2 Methods

The process of this meta-analysis was based on the Preferred Reporting Items for Systematic Reviews and Meta-Analyses (PRISMA) principles (18). The project design was registered in PROSPERO (CRD42025642602).

### 2.1 Search strategy, study selection, and inclusion criteria

Two members of the research team completed the search and selection independently, and a third member joined the process to resolve any disagreements. We searched the PubMed, Embase, and Web of Science databases for articles published through 23 July 2024. The search terms included “urothelial cancer” or “urothelial carcinoma” or “bladder cancer” or “bladder carcinoma” combined with “tumor mutational burden” or “tumor mutation burden” or “tumor mutational load” or “tumor mutation load” or “TMB” or “TML”. The search strategies are listed in **Supplementary Table 1**.

After combining the search results of the three databases, we removed the duplicate records, reviews, case reports, editorial letters, and non-clinical studies. Then, we screened the studies related to TMB and UC by browsing the titles and abstracts. We searched and reviewed the full text of the studies that met the relevance of the topics. Finally, the studies included in this meta-analysis had to meet the following criteria for inclusion: 1) Patients had a definite pathologic diagnosis of urothelial carcinoma. 2) Patients were divided into two cohorts according to TMB level: the trial cohort had a high TMB level, and the control cohort had a low TMB level. 3) The efficacy of ICIs for UC patients was evaluated by the clinical endpoints of OS, PFS, or ORR. (4) The results provided the hazard ratio (HR) and its 95% confidence interval (95% CI) or the Kaplan–Meier curve for survival outcome in the trial cohort versus the control cohort. (5) The results provided the odds ratio (OR) and its 95% CI for ORR, or the number of patients with tumor remission in the trial cohort versus the control cohort.

### 2.2 Data extraction and quality evaluation

The basic information about the studies and vital data used for statistical analysis were extracted, including first author, year of publication, region, type of study, sample size, experimental drug, lines of treatment, sample source of TMB, detection method of TMB, cutoff value of TMB, median TMB value and its range, and clinical outcome.

The Newcastle–Ottawa Scale (NOS) quality assessment scale was suitable for cohort studies. We assessed the quality of the included studies in terms of selection, comparability, and outcome with a total score of 0–9. A score of 7–9 was low risk of bias, a score of 4–6 was high risk of bias, and a score of 0–3 was very high risk of bias (19).

### 2.3 Statistical analysis

Meta-analyses were conducted via the STATA 14.0 software and visualized using forest plots. The statistical difference was significant when the *p*-value <0.05. Heterogeneity was quantified using the I-square and chi-square tests. When *I* ^2^ < 50% or *p* > 0.1, the heterogeneity between studies was low, and the meta-analysis was calculated using the fixed-effects model; otherwise, the random-effects model was used (20). The subgroup analyses were performed on four aspects: region, regimen of immunotherapy, detection method of TMB, and threshold value of TMB. The sensitivity analysis was evaluated to check the stability of the meta-analysis results. Publication bias was visualized by a funnel plot and quantified using Egger’s test and Begg’s test (21, 22).

## 3 Results

### 3.1 Screening results

**Figure 1:**
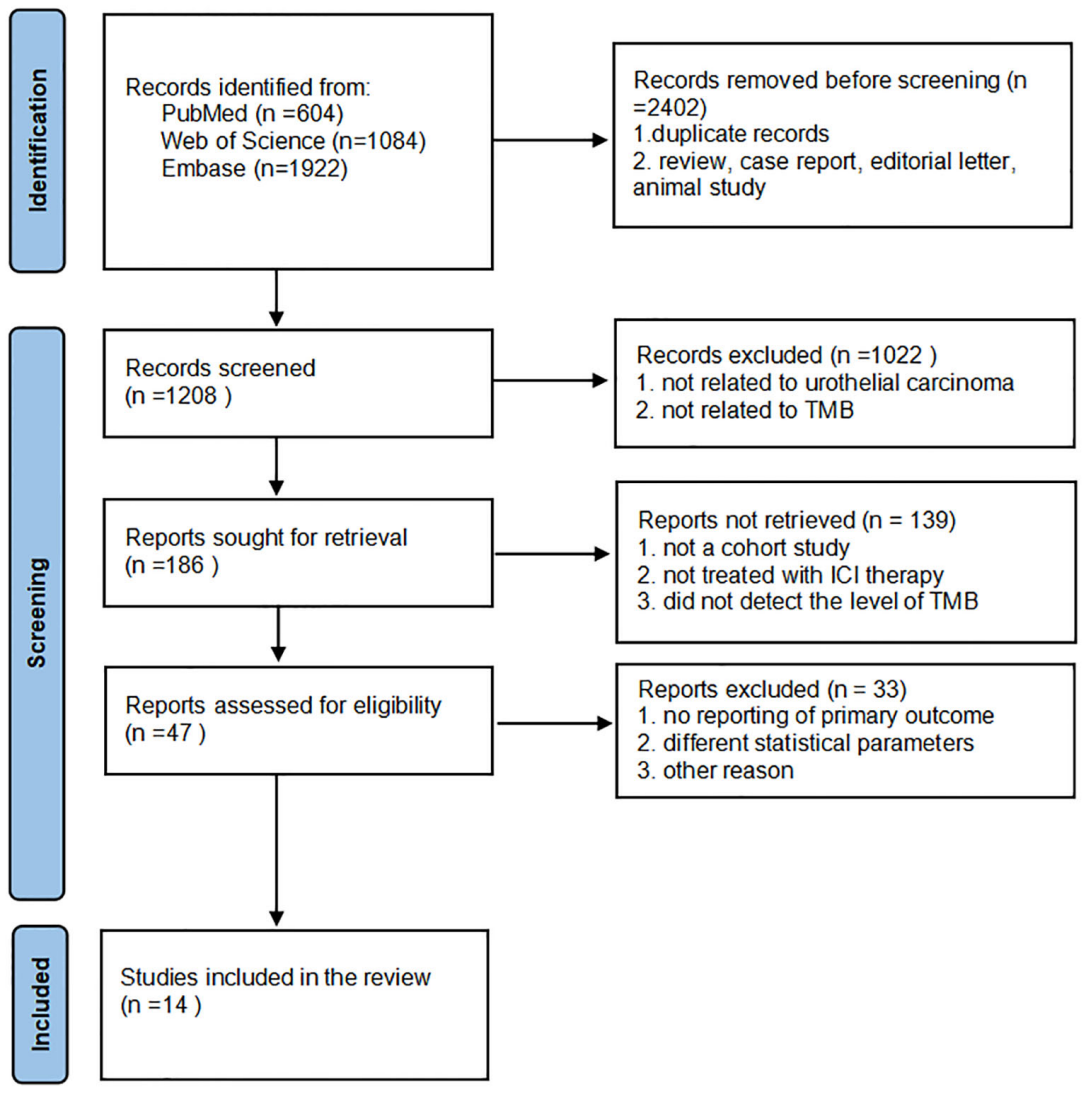
Flowchart diagram of the literature search and screening process.

The screening process is plotted in **Figure 1**. Using the search terms, we collected 2,402 studies from PubMed, Web of Science, and Embase databases. After removing duplicates, reviews, editorial letters, case reports, and animal trials, we assigned 1,208 studies to the screening step. We strictly adhered to the inclusion criteria and then selected 186 eligible reports. We reviewed the titles and abstracts of these studies with reference to the inclusion criteria and selected 47 eligible studies for full review. Finally, we included 14 studies in our meta-analysis.

### 3.2 Study characteristics and quality assessment

The key features extracted from the 14 studies are listed in **Table 1**. The 14 studies included 2,499 patients from the United States, China, and other geographical areas. Six studies included patients with advanced UC, and five studies included patients with metastatic UC. The number of patients with metastatic UC was 1,165 (46.6%), and the number of patients with urothelial carcinoma of the upper urinary tract was 203 (8.1%). Eleven studies were retrospective trials, and three studies were phase II trials. The meta-analysis data were derived from cohort studies of biomarkers in these trials. Outcome data were reported as OS in 13 studies, as PFS in nine studies, and as ORR in seven studies. The experimental drugs included a variety of approved ICIs, with monotherapy in six studies and combination therapy in one study. Therapeutic strategies for advanced UC included first-line and second-line treatment. TMB levels in tumor tissue or blood samples were determined by next-generation sequencing (NGS) or whole-exome sequencing (WES), with the exception of one study that assessed them by targeted exome sequencing analysis. The median TMB level ranged from 1.6 to 113 Mut/Mb. The TMB cutoff value was determined to be 10 Mut/Mb in eleven studies, 1.6 Mut/Mb in one study, and 113 Mut/Mb in another study.

**Table 1:** The main characteristics of the studies included in the meta-analysis.

Author	Year	Region	Type of study	Number of patients (high/low TMB)	Experimental drug	Outcome	Sample source	TMB detection method	TMB cutoff value (Mut/Mb)	Median/average TMB (range)	Lines of treatment
Bakaloudi (23)	2024	United States	Retrospective	339 (116/223)	ICIs	OS, PFS, ORR	NA	NGS	10	NA	1L, 2L+
Alam (24)	2024	United States	Retrospective	160	ICIs	OS, PFS	NA	Targeted exome sequencing	10	52.7 (IQR 39.7, 63.2)	NA
Galffy (25)	2023	Multiple countries	Phase II	15 (8/7)	Avelumab	PFS, ORR	Tissue	WES	1.6	1.6 (0.4–17.7)	1L
Reyes (26)	2023	United States	Retrospective	85 (47/38)	ICIs	OS, PFS, ORR	NA	NGS	10	NA	1L, 2L+
Scobie (27)	2023	United States	Retrospective	332 (130/202)	Atezolizumab, avelumab, cemiplimab, durvalumab, ipilimumab, nivolumab, pembrolizumab	OS	Tissue	NGS	10	8 (4–13)	NA
Bellmunt (28)	2022	Multiple countries	Retrospective	182 (53/129)	Pembrolizumab	OS	Tissue	WES	10	NA	2L
Chawla (29)	2022	United States	Retrospective	113	ICIs	OS, PFS, ORR	Tissue	WES	10	NA	1L, 2L
Graf (30)	2022	United States	Retrospective	245 (83/162)	Pembrolizumab, atezolizumab, nivolumab, durvalumab, avelumab	OS, PFS	Tissue	NGS	10	7.5 (IQR 3.8, 12.5)	1L
Natesan (31)	2022	United States	Retrospective	22 (9/13)	Pembrolizumab, atezolizumab, nivolumab, ipilimumab, durvalumab, tremelimumab	OS, PFS, ORR	Tissue	NGS	10	NA	NA
Sheng (32)	2022	China	Phase II	135 (27/108)	Toripalimab	OS, PFS, ORR	Tissue	WES	10	4.1	2L
Szabados (33)	2022	United States	Retrospective	118 (42/76)	ICIs	OS	Tissue	NGS	10	7.67 (3.75–11.25)	1L
Voutsadakis (34)	2022	Multiple countries	Retrospective	411 (107/304)	ICIs	OS	NA	WES	10	H-TMB: 19.4; L-TMB: 4.6	NA
Rousseau (35)	2021	United States	Retrospective	203 (77/126)	ICIs	OS	Blood	NGS	NA	NA	NA
Galsky (36)	2020	Multiple countries	Phase II	139 (70/69)	Nivolumab	OS, PFS, ORR	Tissue	WES	113	113	2L+

ICIs, immune checkpoint inhibitors; NGS, next-generation sequencing; WES, whole-exome sequencing; IQR, interquartile range; TMB, tumor mutation burden; OS, overall survival; PFS, progression-free survival; ORR, objective response rate.

Based on the NOS assessment, nine studies were evaluated as being high quality, and only one study was rated as medium quality (**Supplementary Table 3**). Four studies were abstract reports and did not qualify for risk of bias assessment. Although the inclusion of these articles may increase the risk, they were still included in the study because of the integrity of the data. These studies, consisting of hundreds of patients, contributed to the pooled results of the meta-analysis.

### 3.3 Main results and assessment of heterogeneity

**Figure 2:**
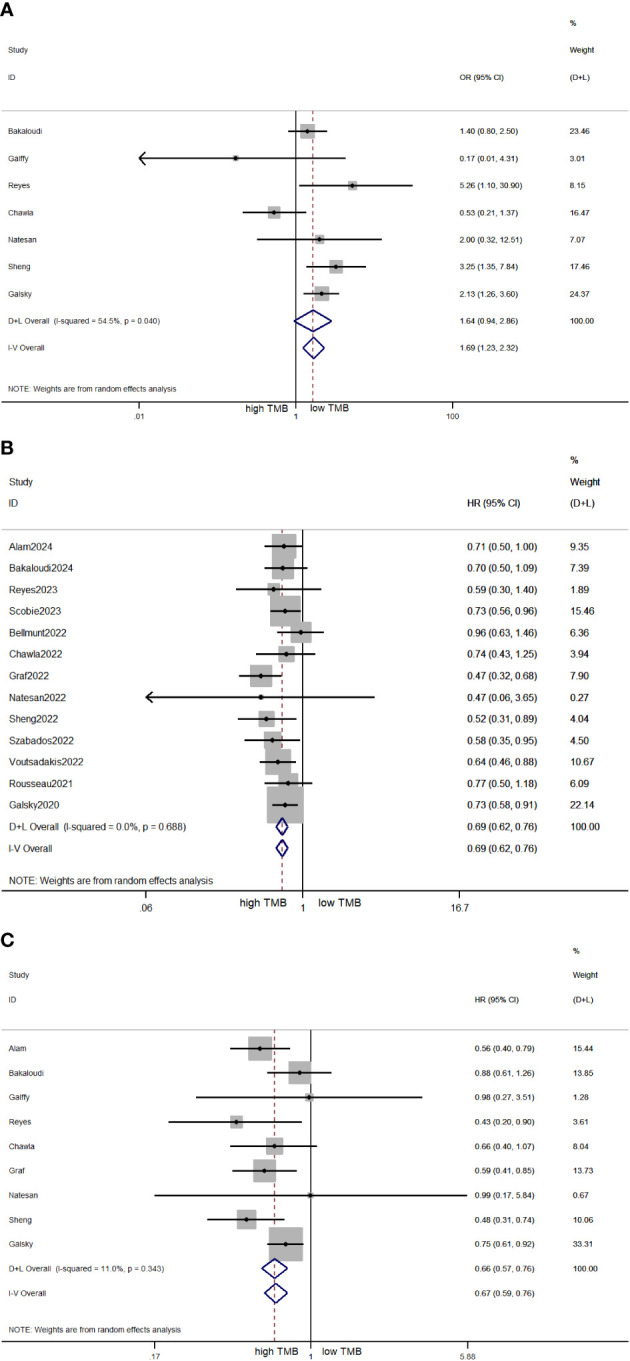
Forest plots of OR for ORR **(A)**, HR for OS **(B)**, and HR for PFS **(C)** in patients with high TMB versus low TMB. OR, odds ratio; ORR, objective response rate; HR, hazard ratio; OS, overall survival; PFS, progression-free survival; TMB, tumor mutational burden.

The pooled effect of OR for ORR was evaluated across seven studies involving 848 patients. The high TMB cohort exhibited a superior ORR with no significant difference from the low TMB cohort (OR = 1.64, 95% CI 0.94, 2.86, *p* = 0.08; **Figure 2A**). The heterogeneity among the included studies was high (*I* ^2^ = 54.5%, *p* = 0.04), so the meta-analysis was conducted under a random effects model. As illustrated in **Figure 2B**, the pooled effect of HR for OS was evaluated across 13 studies involving 2,484 patients. The pooled effect of HR for OS was 0.69 (95% CI 0.62, 0.76, *p* < 0.05), and the heterogeneity was low (*I* ^2^ = 0%, *p* = 0.688). The meta-analysis result indicated that patients expressing high TMB levels had significantly better OS. As illustrated in **Figure 2C**, there were nine studies involving 1,253 patients to calculate the pooled effect of HR for PFS. The pooled effect of HR for PFS was 0.67 (95% CI 0.59, 0.76, *p* < 0.05), and the heterogeneity was low (*I* ^2^ = 11%, *p* = 0.343). Similarly, patients expressing high TMB levels had significantly better PFS.

### 3.4 Subgroup analysis

On account of the high heterogeneity in the ORR meta-analysis, the subgroup analysis was performed to explore the relevant factors affecting the heterogeneity. The results of the subgroup analyses are shown in **Supplementary Figures 1–4**. There was no further improvement in heterogeneity after subgroup analysis based on four related factors. The survival analyses for OS and PFS had low heterogeneity, so we only proceeded with the subgroup analysis based on the TMB threshold value (**Supplementary Figures 5, 6**). In the 10 Mut/Mb cutoff subgroup, the pooled effect of HR for OS was 0.67 (95% CI 0.59, 0.75, *p* < 0.05), and for PFS, it was 0.62 (95% CI 0.52, 0.73, *p* < 0.05). The heterogeneity in these subgroups remained low.

### 3.5 Sensitivity analysis and publication bias

**Figure 3:**
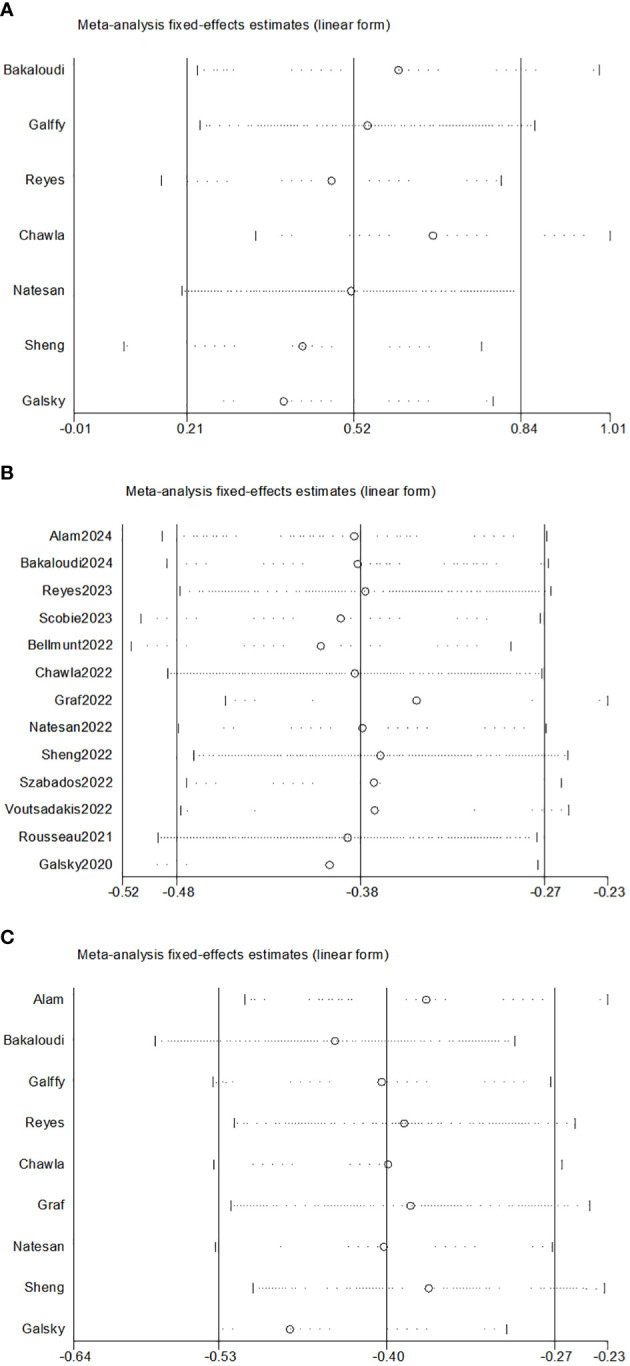
Sensitivity analysis of pooled effects for ORR **(A)**, OS **(B)**, and PFS **(C)**. ORR, objective response rate; OS, overall survival; PFS, progression-free survival.

**Figure 4:**
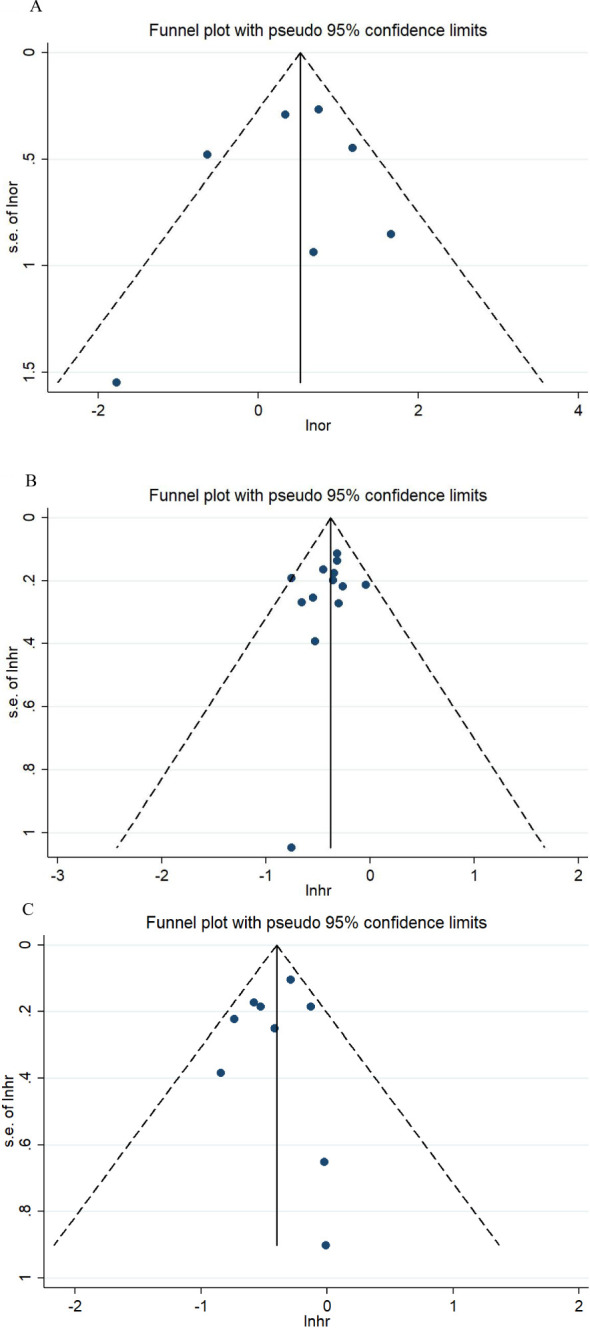
Funnel plots for ORR **(A)**, OS **(B)**, and PFS **(C)**. ORR, objective response rate; OS, overall survival; PFS, progression-free survival.

As illustrated in **Figure 3**, the sensitivity analysis showed no intense alteration of the pooled effects in meta-analysis after excluding each article in turn. Thus, the results of the meta-analyses were stable and reliable by the random effects model. The funnel plots for ORR, OS, and PFS were intuitively symmetrical (**Figure 4**). Combined with the results of Begg’s and Egger’s tests, there was no proof of publication bias in the ORR, OS, and PFS analyses (Begg’s test: *p* = 0.548, *p* = 0.360, *p* = 0.917; Egger’s test: *p* = 0.261, *p* = 0.065, *p* = 0.069).

## 4 Discussion

This study is the first meta-analysis to evaluate the predictive value of TMB for response and survival benefit in patients with UC treated with ICIs. The results suggested that ICIs provided superior survival for patients with UC in the high TMB group compared to the low TMB group. After ICI therapy, the risk of death in UC patients with high TMB decreased by 31% compared to patients with low TMB.

Decades of research in molecular oncology and gene sequencing have identified genetic mutations in tumor cells that are related to the occurrence and development of multiple malignancies. TMB, as an aggregation of somatic mutations, provides a measure to quantify the aberrant tumor genome. A series of retrospective and prospective research studies have been conducted to identify TMB as a predictor of ICIs. The most important study was the Keynote-158 trial, which promoted the expansion of treatment options for patients with high TMB tumors (37). Sample sizes of most tumor types were small in this trial, and urothelial carcinoma was not included. Analysis of survival endpoints, meanwhile, was lacking. Whether TMB status could predict the survival benefit of ICIs in patients with urothelial carcinoma remains a hotly debated topic.

Data from a real-world study demonstrated that metastatic UC patients with TMB ≥ 10 Mut/Mb treated with single-agent ICIs as first-line therapy had more favorable PFS and OS (30). The results from another real-world cohort study showed that higher TMB was associated with longer OS, although the difference was not statistically significant (23). In the first-line subset, a comparison of patients with high TMB versus low TMB also did not show a promising advantage. The phase II trial focusing on correlative biomarkers of toripalimab demonstrated prolonged PFS and OS in high TMB UC patients with previous treatment (32). The authors considered TMB as an independent biomarker for immunotherapy in UC patients who experienced a failure of standard therapy. The CheckMate 275 trial, which explored the TMB biomarker of nivolumab for patients expressing a positive response and survival, has made sustained progress (36).

TMB is a continuous variable that leads to inherent challenges in clinical categorization. Whether the definitive cutoff (10 Mut/Mb) provided by the Keynote-158 trial is optimal and equally valid across all cancer types warrants further investigation. A study from MSK-IMPACT TMB data demonstrated that the TMB value fluctuated widely among 25 eligible tumors, and the correlation between TMB and ORR gradually increased and then leveled off with increasing TMB (38). The investigators identified the structural breakpoint of 10 Mut/Mb as the optimal cutoff for a high TMB level. Considering the inconsistent thresholds selected by the included studies, which introduced heterogeneity in the results, we conducted the subgroup analysis for the cutoff value 10 Mut/Mb and other thresholds. The subgroup analysis indicated that the benefit in the high TMB group was still established in the 10 Mut/Mb cutoff subgroup. These results may pave the way for further validation of 10 Mut/Mb as a specific TMB cutoff for UC patients to derive benefit from ICIs.

In addition to a fixed value of 10 Mut/Mb, some researchers have suggested that applying a higher or tumor-specific threshold may be more appropriate to replace a single fixed threshold for the stratification of patients to benefit from ICIs (39). After examining the TMB values of a specific tumor patient population, selecting the median or quartile of TMB values as a threshold is also an applicable approach. Utilizing the continuous variable characteristic of TMB, plotting continuous relationship curves for TMB and efficacy can guide clinical decision-makers to make flexible determinations (40). Moreover, there are a variety of statistical methods that enable non-linear evaluation of TMB without the use of cutoffs (41–43). High-quality statistical software applications can provide more assessment methods for TMB to predict the efficacy of ICIs.

At present, WES and NGS are the main detection methods for TMB in clinical settings. The method of WES was to detect the sequencing of matched tumor and non-malignant genomes and then directly provide the number of missense mutations (44). Alternatively, NGS only detected tumor DNA using panel sequencing and reported the unit of TMB as Mut/Mb. Compared to the direct measurement of TMB, panel-based sequencing introduced a stochastic error by subsampling the target sequence. To the advantage of decreasing costs, NGS was still driving the feasibility of addressing clinical needs. Among the reports included in our study, eight studies detected TMB using NGS, while six used WES. Including additional types of non-synonymous mutations and excluding known oncogenic variants in the calculating process both contributed to improving the accuracy of TMB measurement (13). As a result, panel-based TMB soon emerged as an elegant way to fix on crowds anticipated to profit from ICIs.

Blood-based TMB (bTMB) is a non-invasive method for detecting circulating tumor DNA in a blood sample that is applicable in situations where there is a lack of adequate tissue for genomic testing. bTMB can profile the full spectrum of tumor-related genomic mutations rather than being limited to a single tumor site, especially in metastases (45). The phase III MYSTIC trial showed that the detection rate of valid bTMB (809/1,001; 81%) was higher compared to tissue-based TMB (tTMB) (460/735; 63%) in metastatic non-small cell lung cancer (NSCLC) (46). Another study assessing bTMB in patients with NSCLC demonstrated that bTMB was consistent with tTMB when it was of high value (47). Plasma samples with low bTMB values may be influenced by insufficient circulating tumor DNA because of earlier stages of disease and low tumor burden. In the phase 2 study of nivolumab, TMB status was evaluated concurrently using tTMB and bTMB in the detection method (48). The results showed that the ORR value for the nivolumab+ipilimumab cohort was 38.6% in high tTMB and 22.5% in high bTMB. Meanwhile, in the nivolumab cohort, the ORR value was 29.8% and 15.6% in patients with tTMB-H and bTMB-H, respectively. Considering the availability of urothelial carcinoma tumor tissue, the majority of the included articles used tumor tissue as TMB samples.

The exploratory analysis of the CheckMate 275 trial demonstrated that the predictive value of TMB combined with PD-L1 for response to nivolumab was better than PD-L1 alone (36). The exploratory analysis for the phase III KEYNOTE-361 study assessed the usefulness of TMB and PD-L1 in predicting response to immunotherapy (49). Continuous TMB value and PD-L1 combined positive score (CPS) had a positive association with the benefit of pembrolizumab monotherapy. Compared with patients with either biomarker alone, patients expressing high TMB and PD-L1 CPS obtained the most benefit from pembrolizumab. The results of the phase II KEYNOTE-052 trial were consistent with a tendency toward a decreased risk of death in the high TMB and PD-L1 CPS subgroup (28). The meta-analysis of the predictive evaluation of PD-L1 for UC patients treated with ICIs showed that PD-L1 may be a favorable biomarker for ORR but not for OS (50). Our meta-analysis found that TMB was suitable for predicting the survival in UC patients treated with ICIs, which may neatly fill the gap. Future randomized trials of ICIs using a combination of TMB and PD-L1 as dual biomarkers to identify the potential benefit group in UC are warranted.

Our study pooled and analyzed the results of existing studies to provide preliminary advice on the predictive value of TMB for immunotherapy in the UC patient population. Nevertheless, several trivial limitations could not be overcome. Confounding factors in the majority of retrospective or non-randomized studies were inevitably introduced into the study process. The survival of UC patients was also affected by their own factors, such as the level of interleukin-8, a key chemokine secreted by tumor cells and immune cells (51). Furthermore, the combination of antihypertensive drugs modulating the renin–angiotensin–aldosterone system increased the efficacy of ICIs, and the combination regimen prolonged the survival of UC patients (52, 53). None of these confounding factors can be eliminated in retrospective or non-randomized studies. Therefore, future prospective clinical trials are desirable to eliminate these confounding factors using rational designs. In addition, the majority of the biomarker analyses were extended studies based on completed clinical trials. The number of patients available for genetic sequencing was small. The lack of trial information and outcomes in specific patient cohorts prevented subgroup analysis of prognosis-related factors such as age and performance status (PS), and analysis adjusted for background factors. The presence of these confounding factors may also have an indelible effect on the results.

With the development of genetic testing technology, more prospective studies on predictive biomarkers for UC immunotherapy will be conducted to further confirm the results of this study. If the same promising results are confirmed in prospective studies, TMB will become an indispensable biomarker for UC patients independent of the other predictive biomarkers, including PD-L1 expression, mismatch repair deficiency, microsatellite instability, and the tumor microenvironment. Even patients with low expression of PD-L1 still have a hope of benefiting from immunotherapy. With the progress of personalized medicine in the field of immunotherapy, the improvement of TMB as a biomarker remains important.

## 5 Conclusions

This first meta-analysis to appraise the prognostic value of TMB in UC patients treated with ICIs demonstrated that more survival benefit was observed in the high TMB group than in the low TMB group at a 10 Mut/Mb cutoff. Hence, we suggest that TMB may be a favorable biomarker to identify UC patients with better efficacy of ICIs. Future prospective clinical trials are required to further validate the applicability of TMB as a predictor for immunotherapy in UC.

## Data Availability

The datasets presented in this study can be found in online repositories. The names of the repository/repositories and accession number(s) can be found in the article/**Supplementary Material**.
