# Supplementary material for: Role of tumor mutational burden in patients with urothelial carcinoma treated with immune checkpoint inhibitors: a systematic review and meta-analysis
Source: Front Immunol. 2025 May 26;16:1592761. doi: 10.3389/fimmu.2025.1592761 (PMC12146348; doi:10.3389/fimmu.2025.1592761)
Supplement: Supplementary file 1 [file DataSheet1.docx]

Supplementary Table 1 The search strategies in databases PubMed, Embase, and Web of Science.

| **Database** | **Query** | **Results** |
| --- | --- | --- |
| PubMed | ((urothelial cancer) OR (urothelial carcinoma) OR (bladder cancer) OR (bladder carcinoma)) AND ((tumor mutational burden) OR (tumor mutation burden) OR (tumor mutational load) OR (tumor mutation load) OR (TMB) OR (TML)) | 604 |
| Embase | ('urothelial cancer'/exp OR 'urothelial cancer' OR (urothelial AND ('cancer'/exp OR cancer)) OR 'urothelial carcinoma'/exp OR 'urothelial carcinoma' OR (urothelial AND ('carcinoma'/exp OR carcinoma)) OR 'bladder cancer'/exp OR 'bladder cancer' OR (('bladder'/exp OR bladder) AND ('cancer'/exp OR cancer)) OR 'bladder carcinoma'/exp OR 'bladder carcinoma' OR (('bladder'/exp OR bladder) AND ('carcinoma'/exp OR carcinoma))) AND ('tumor mutational burden'/exp OR 'tumor mutational burden' OR (('tumor'/exp OR tumor) AND mutational AND ('burden'/exp OR burden)) OR 'tumor mutation burden'/exp OR 'tumor mutation burden' OR (('tumor'/exp OR tumor) AND ('mutation'/exp OR mutation) AND ('burden'/exp OR burden)) OR 'tumor mutational load' OR (('tumor'/exp OR tumor) AND mutational AND ('load'/exp OR load)) OR 'tumor mutation load'/exp OR 'tumor mutation load' OR (('tumor'/exp OR tumor) AND ('mutation'/exp OR mutation) AND ('load'/exp OR load)) OR tmb OR tml) | 1922 |
| Web of Science | ((((TS=(urothelial cancer)) OR TS=(urothelial carcinoma)) OR TS=(bladder cancer)) OR TS=(bladder carcinoma))AND ((((((TS=(tumor mutational burden)) OR TS=(tumor mutation burden)) OR TS=(tumor mutational load)) OR TS=(tumor mutation load)) OR TS=(TMB)) OR TS=(TML)) | 1084 |

Supplementary Table 2: The additional information of included studies.

| **Author** | **Year** | **Age, year**  **median(range)** | **Sex,**  **male/female** | **PS** | **Stage of UC(mUC%)** | **Upper urinary tract,%** | **Regimen of immunotherapy** |
| --- | --- | --- | --- | --- | --- | --- | --- |
| Bakaloudi  [16] | 2024 | 69 | 263/79 | 0-1+ | aUC(12) | 16 | NA |
| Alam[17] | 2024 | NA | NA | NA | mUC | NA | NA |
| Galffy[18] | 2023 | 71.0 (51.0-83.0) | 12 /8 | 0-2 | aUC(85) | NA | combination with target drug |
| Reyes[19] | 2023 | NA | NA | NA | mUC | NA | monotherapy |
| Scobie[20] | 2023 | 70.3 (60.8-79.8) | 329/3 | NA | UC | 10.8 | NA |
| Bellmunt[21] | 2022 | 67 (29-88) | 135/47 | 0-2 | aUC(31.4) | 17.8 | monotherapy |
| Chawla[22] | 2022 | NA | NA | 0-2 | aUC(55) | 20 | NA |
| Graf[23] | 2022 | NA | NA | 0-3+ | mUC | 22.8 | monotherapy |
| Natesan[24] | 2022 | NA | NA | NA | aUC | NA | NA |
| Sheng[25] | 2022 | NA | NA | NA | mUC | NA | monotherapy |
| Szabados[26] | 2022 | 74.5 (68–82) | 87/31 | 0-1+ | mUC | NA | monotherapy |
| Voutsadakis  [27] | 2022 | 68.1 | 303/108 | NA | UBC(33.1) | - | NA |
| Rousseau  [28] | 2021 | NA | NA | NA | UC | NA | NA |
| Galsky[29] | 2020 | 64.5 (38–85) | 109/30 | NA | aUC(80.6) | NA | monotherapy |

UC: urothelial carcinoma; aUC: advanced urothelial carcinoma; mUC: metastatic urothelial carcinoma；UBC: urothelial bladder carcinomas;

~~Supplementary Table 1~~ Supplementary Table 3 The Newcastle-Ottawa Scale (NOS) assessment for risk of bias.

| Study | Selection | Comparability | Outcome | Total score∗ |
| --- | --- | --- | --- | --- |
| Galffy | 4 | 0 | 3 | 7 |
| Scobie | 4 | 0 | 3 | 7 |
| Bellmunt | 4 | 0 | 3 | 7 |
| Chawla | 4 | 0 | 3 | 7 |
| Graf | 4 | 0 | 3 | 7 |
| Natesan | 4 | 0 | 2 | 6 |
| Sheng | 4 | 0 | 3 | 7 |
| Szabados | 4 | 0 | 3 | 7 |
| Voutsadakis | 4 | 2 | 3 | 9 |
| Galsky | 4 | 1 | 2 | 7 |

∗NOS points: 0 to 3: very high risk of bias; 4 to 6: high risk of bias; 7 to 9: low risk of bias


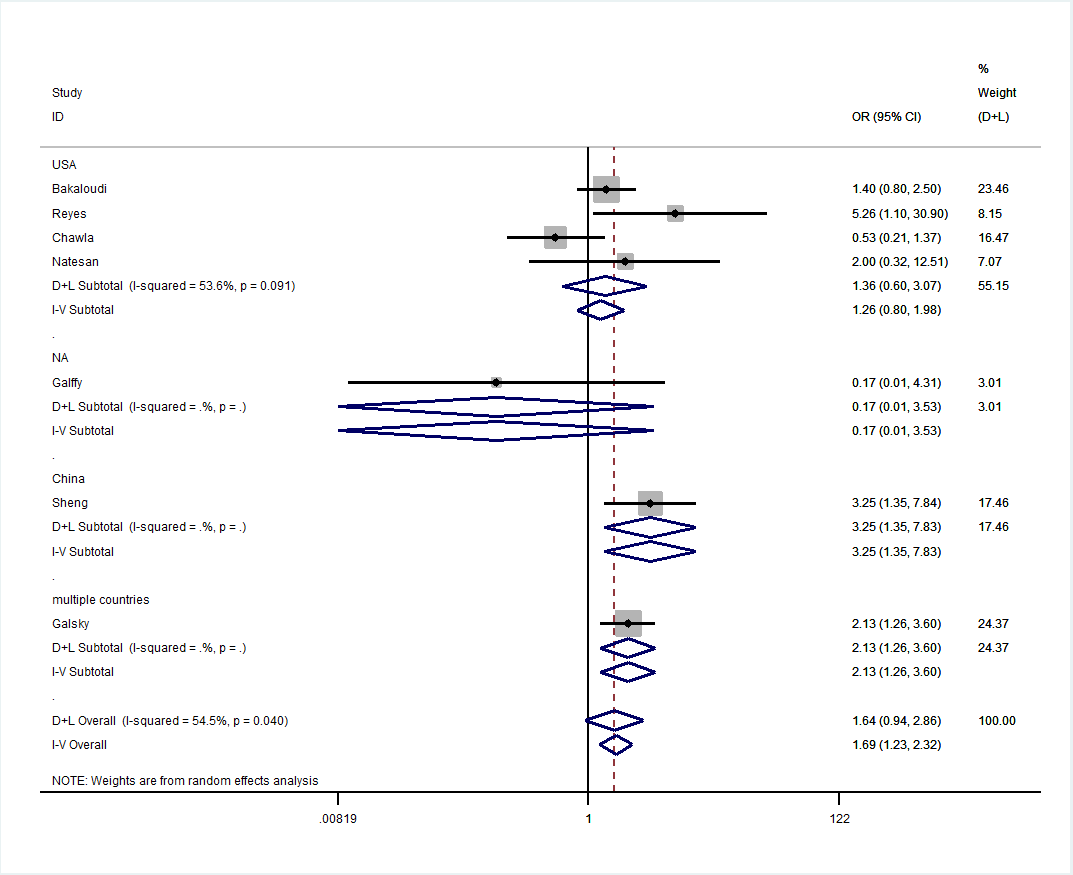


Supplementary figure 1 Forest plots of subgroup analysis for ORR by region.


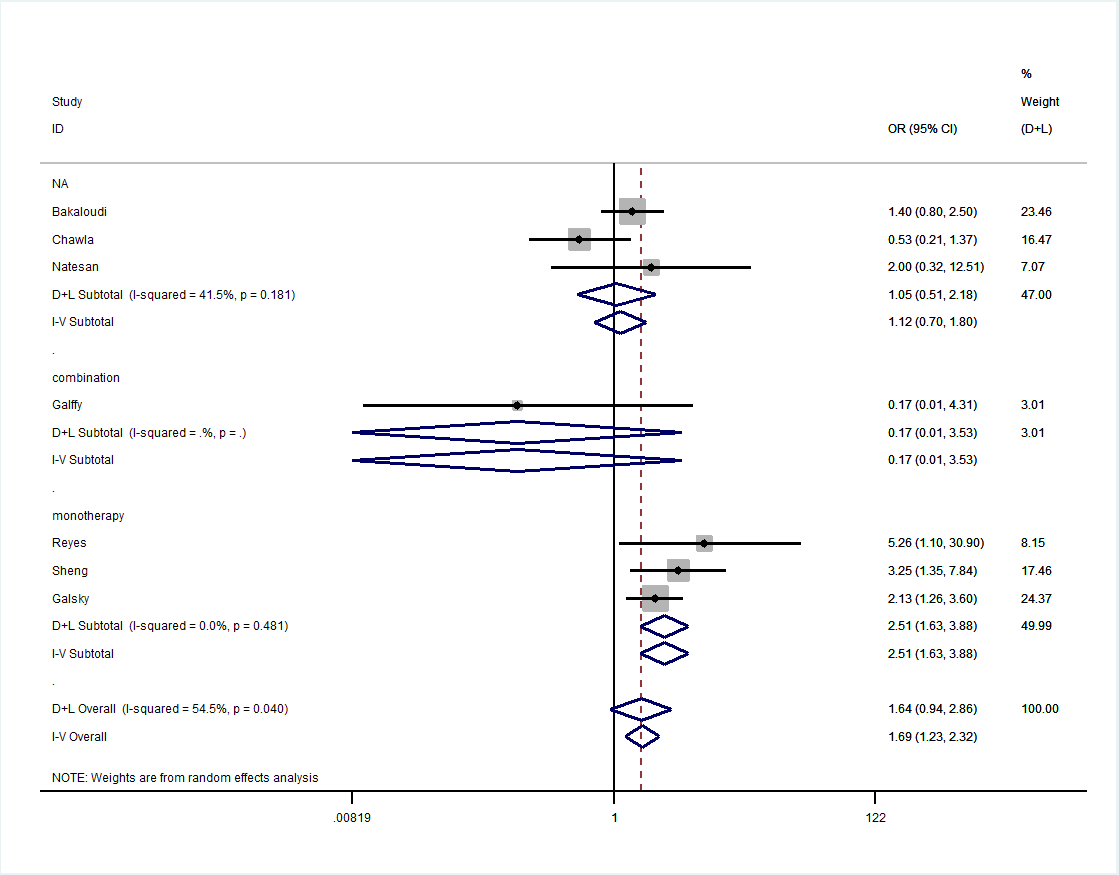


Supplementary figure 2 Forest plots of subgroup analysis for ORR by regimen of immunotherapy.


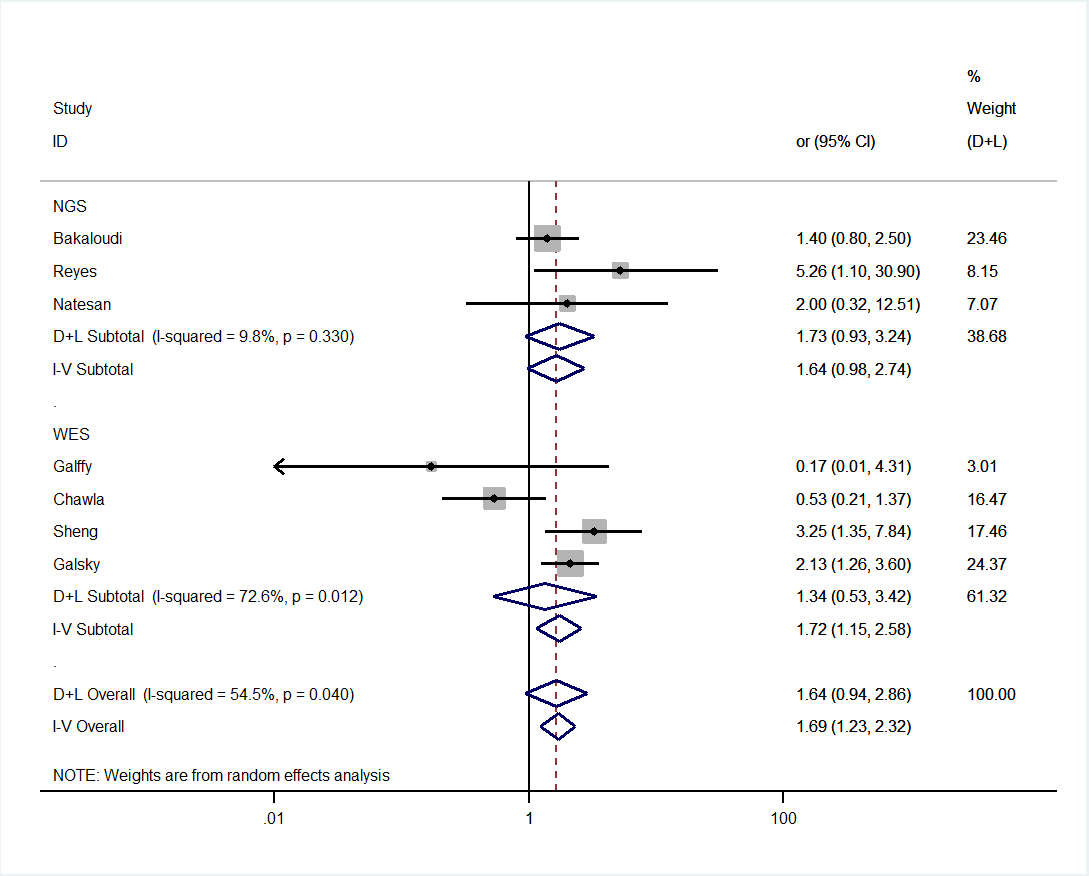


Supplementary figure 3 Forest plots of subgroup analysis for ORR by TMB detection method.


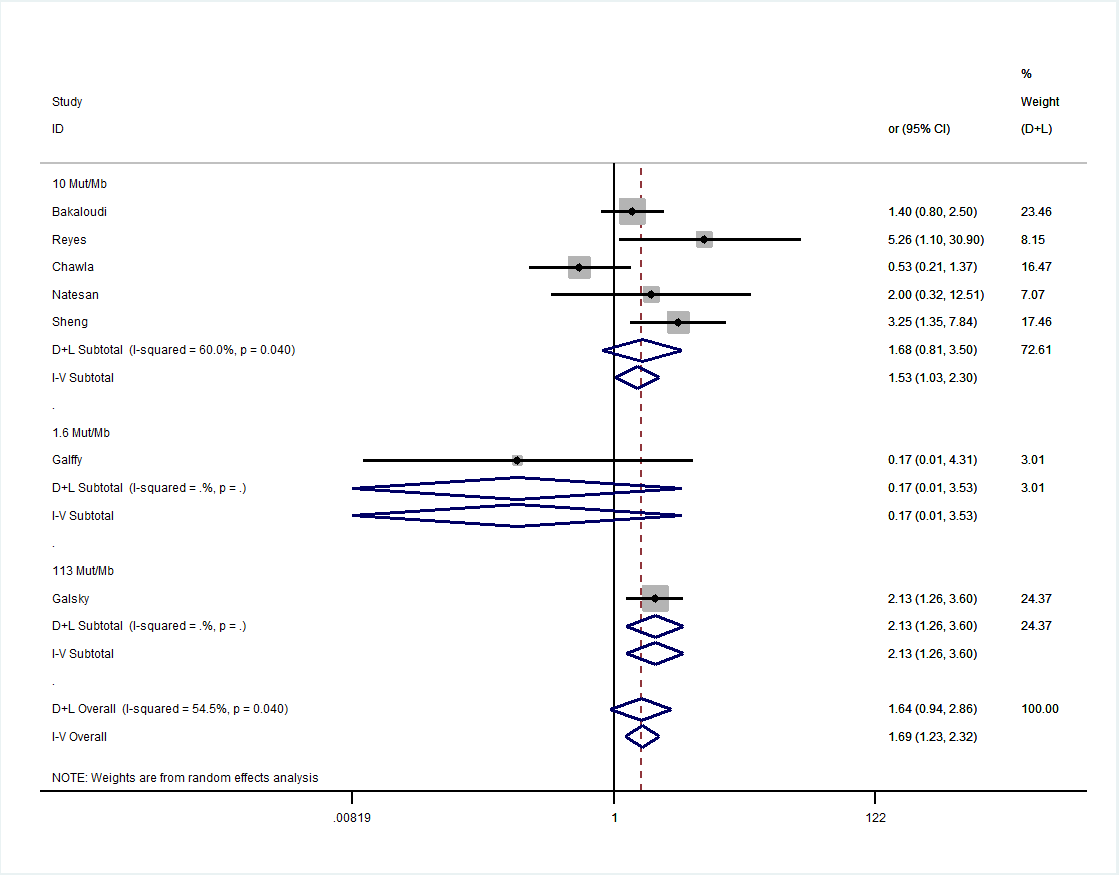


Supplementary figure 4 Forest plots of subgroup analysis for ORR by TMB cutoff value.


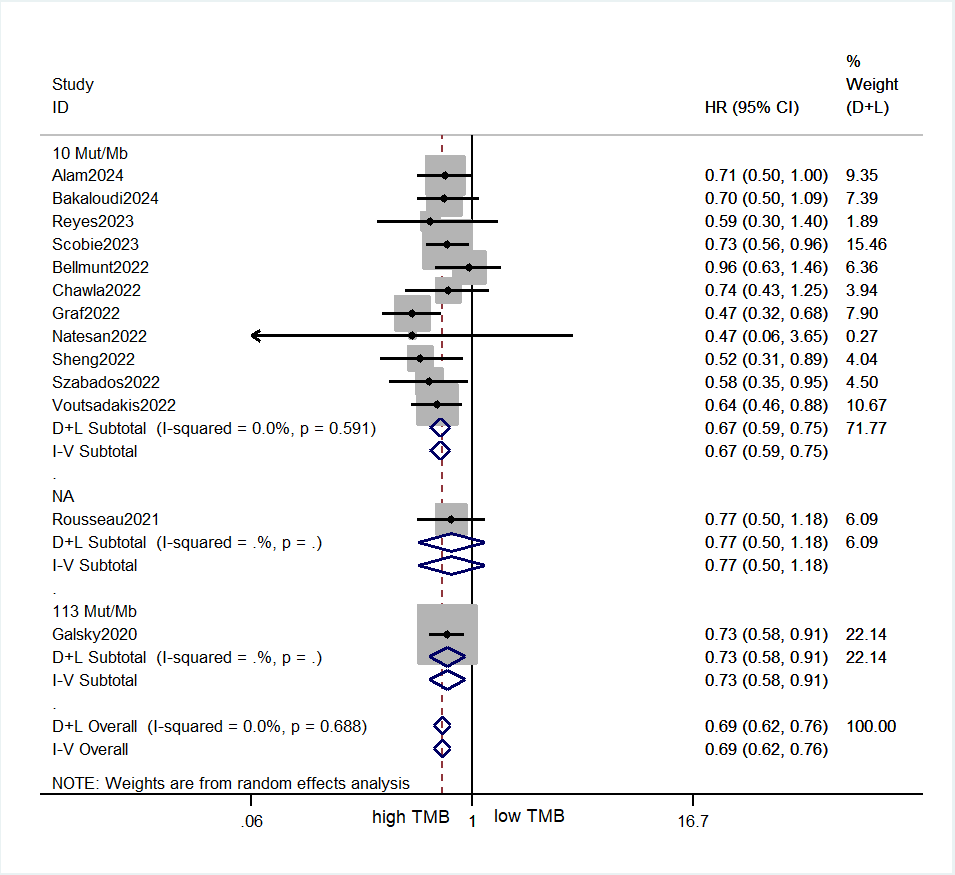


Supplementary figure 5 Forest plots of subgroup analysis for OS by TMB cutoff value.


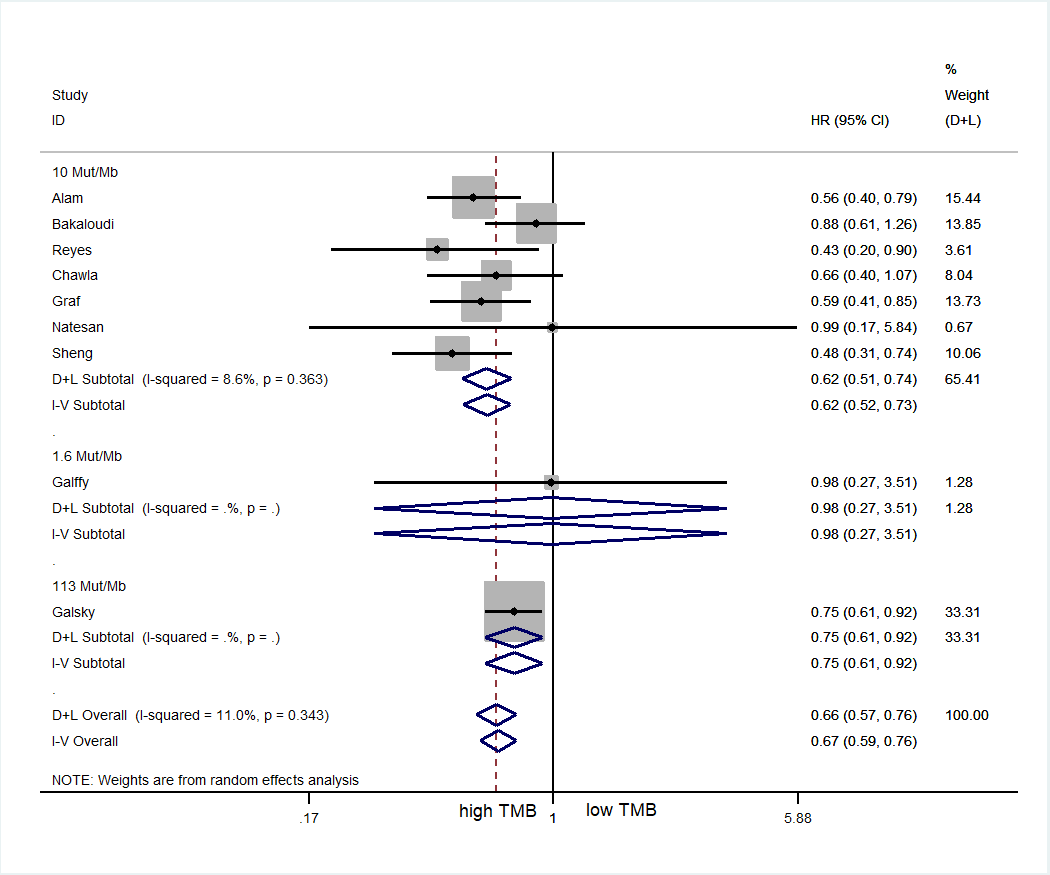


Supplementary figure 6 Forest plots of subgroup analysis for PFS by TMB cutoff value.
